# Cervical Antibodies to Herpes Simplex Virus Proteins in Pregnancy and Puerperium: A Pilot Study

**DOI:** 10.1155/S1064744996000038

**Published:** 1996

**Authors:** D. Heather Watts, Jeanne-Marie Guise, Zane Brown, Lawrence Corey, Rhoda L. Ashley

**Affiliations:** 1 Department of Obstetrics and Gynecology University of Washington School of Medicine 1959 NE Pacific Street RH-20 Seattle WA 98195 USA; 2 Department of Laboratory Medicine University of Washington School of Medicine Seattle WA USA; 3 Department of Medicine University of Washington School of Medicine Seattle WA USA; 4 Department of Microbiology University of Washington School of Medicine Seattle WA USA; 5 Department of Obstetrics and Gynecology CB7570 University of North Carolina Chapel Hill NC 27599-7570 USA

## Abstract

*Objective:* This study was undertaken to evaluate the changes in total and anti-herpes simplex virus (HSV)-specific cervical IgA and IgG antibody profiles during and after pregnancy.

*Methods:* Serum and cervical secretions were obtained from pregnant patients before 20 weeks gestation, at 34–36 weeks gestation, and at 6 weeks postpartum and tested for total IgA and IgG antibody and for IgA and IgG to HSV proteins by Western blot.

*Results:* Seven women were HSV seronegative, 14 HSV-1 seropositive, and 14 HSV-2 ± HSV-1 seropositive. Minimal changes in the serum anti-HSV profiles were seen over the 3 visits. The total cervical IgA, IgG, and protein levels did not change between the 2 pregnancy visits but tended to increase at the postpartum visit. No consistent change in cervical HSV-specific IgA and IgG was seen during pregnancy, but the levels increased markedly at the postpartum visit.

*Conclusions:* Lower cervical anti-HSV antibody levels may be related to the previously reported increased frequency of a reactivation of HSV during late pregnancy. Further evaluation is necessary to confirm and quantify the changes in genital immunity during pregnancy and to evaluate whether the increased levels at the postpartum visit are sustained.


Infectious Diseases in Obstetrics and Gynecology 4:7–15 (1996)
© 1996 Wiley-Liss, Inc.

### Cervical Antibodies to Herpes Simplex Virus Proteins in Pregnancy and Puerperium: A Pilot Study

**D. Heather Watts, Jeanne-Marie Guise, Zane Brown, Lawrence Corey, and Rhoda L. Ashley**

*Departments of Obstetrics and Gynecology (D.H.W., J.-M.G., Z.B.), Laboratory Medicine (L.C., R.L.A.), Medicine (L.C.), and Microbiology (R.L.A.), University of Washington School of Medicine, Seattle, WA*

ABSTRACT

***Objective:*** **This study was undertaken to evaluate the changes in total and anti-herpes simplex virus (HSV)-specific cervical IgA and IgG antibody profiles during and after pregnancy.**

***Methods:*** **Serum and cervical secretions were obtained from pregnant patients before 20 weeks gestation, at 34–36 weeks gestation, and at 6 weeks postpartum and tested for total IgA and IgG antibody and for IgA and IgG to HSV proteins by Western blot.**

***Results:*** **Seven women were HSV seronegative, 14 HSV-1 seropositive, and 14 HSV-2 ± HSV-1 seropositive. Minimal changes in the serum anti-HSV profiles were seen over the 3 visits. The total cervical IgA, IgG, and protein levels did not change between the 2 pregnancy visits but tended to increase at the postpartum visit. No consistent change in cervical HSV-specific IgA and IgG was seen during pregnancy, but the levels increased markedly at the postpartum visit.**

***Conclusions:*** **Lower cervical anti-HSV antibody levels may be related to the previously reported increased frequency of a reactivation of HSV during late pregnancy. Further evaluation is necessary to confirm and quantify the changes in genital immunity during pregnancy and to evaluate whether the increased levels at the postpartum visit are sustained.** © 1996 Wiley-Liss, Inc.

KEY WORDS

Local immunity, IgA, IgG, genital infection, Western blot

Genital herpes simplex virus (HSV) infections are common among pregnant women, with up to 40% having antibody to HSV-2.[1-4] The risk factors for the perinatal transmission of HSV include recent primary genital infection, cervical rather than only labial HSV shedding, use of fetal-scalp electrodes, and lack of maternal antibody, particularly antibody to an HSV-2 type-specific glycoprotein, gG.[5-9] Among some women with recurrent genital HSV infections, reactivation increases in the third trimester,[10] which may result in a greater risk of perinatal transmission. The factors responsible for the increased rate of reactivation in late pregnancy have not been elucidated, but may be associated with the immunologic changes characteristic of late pregnancy.[11,12]

One possible contributor to the increased genital HSV reactivation in late pregnancy is a change in local antibody levels. In addition to serum antibody, a genital HSV infection induces mucosal antibodies,[13-19] which are directed against a number of viral proteins, that are demonstrable months after the infection and neutralize HSV in vitro. Although systemic levels of immunoglobulins do not appear to

**Address correspondence/reprint requests to Dr. D. Heather Watts, Department of Obstetrics and Gynecology, University of Washington, 1959 NE Pacific Street, RH-20, Seattle, WA 98195.**

**Dr. Jeanne-Marie Guise is now at Department of Obstetrics and Gynecology, CB7570, University of North Carolina, Chapel Hill, NC 27599-7570.**

Received November 24, 1995
Accepted February 26, 1996

change during pregnancy,[20] the effect of pregnancy on mucosal antibodies has not been assessed. To evaluate the effects of pregnancy on circulating and local cervical total and anti-HSV-specific immunoglobulins, we used quantitative assays to assess cervical total IgA and IgG levels and semiquantitative methods to analyze the HSV-specific IgA and IgG in serum and cervical secretions collected during the first half of pregnancy, at 34–36 weeks gestation, and at 6 weeks postpartum.

### SUBJECTS AND METHODS

### Study Population and Protocol

Pregnant women were enrolled in the study from the prenatal clinics of the University of Washington Medical Center and Harborview Medical Center between January 1991 and March 1992. All subjects gave informed consents as approved by the University of Washington Institutional Review Board. The patients were enrolled before 20 weeks gestation and were seen again at 34–36 weeks gestation and 6 weeks postpartum. Of the 55 patients who were enrolled, 37 completed at least 2 visits. Of the 18 (33%) who did not complete the study, 13 were lost to follow-up, 1 moved from the area, and 4 were removed for obstetric reasons (spontaneous abortion in 3 and placenta previa in 1). All women had blood drawn at entry (8–20 weeks) to determine their HSV serologic statuses. Herpes viral cultures of the labia and cervix were performed, and cervical secretions were sampled at all visits. A brief questionnaire regarding current symptoms of a genital or oral HSV infection was completed at each visit. Viral cultures were performed as previously described. The serum specimens were assayed for IgA and IgG antibodies to HSV-1 and HSV-2 by Western blot analyses.[21,22]

### Cervical Secretions

The cervical secretions were obtained as previously described.[18] Briefly, a vaginal speculum was inserted and 2 ophthalmic tear-flow test strips ("Sno-strip," Akorn, Inc., Abita Springs, LA) were placed sequentially on the cervical os until they were saturated to the shoulder, approximately 10 sec each. This technique provided a constant volume of secretions with each sample (mean volume = 357 ± 77 μl/sample).[18] The combined papers from a single sampling were trimmed at the shoulder, and the saturated strips were then sealed in sterile vials containing 500 μl of a 0.01 sodium azide solution in phosphate-buffered saline (PBS) and frozen at −20°C.

### Specimen Preparation

The samples were thawed. After the Sno-strips were removed, the vials were vortexed, then centrifuged at 13,960 *g* for 15 min. The supernatant volumes were recorded, and a 50-μl portion was removed from each sample for protein analysis (Bio-Rad, Richmond, CA) as previously described.[18] The samples were tested for occult blood by Hemoccult (Smith-Kline Diagnostics, San Jose, CA), but the positive samples were not analyzed.

### Total Cervical IgG, IgA Determinations

Purified human IgA (Sigma, St. Louis, MO) and human IgG standards (Pierce, Rockford, Il) were serially diluted 2-fold to 1:10,000 and 1:80,000, respectively, in carbonate buffer (pH 9.6) and dispensed into 96-well plates. The secretions were serially diluted 2-fold from 1:40 for IgA and from 1:200 for IgG into the same plates. Bound antibodies were detected with peroxidase-conjugated goat anti-human α chain or γ chain (Tago, Burlingame, CA) and 3,3′,5,5′-tetramethylbenzidine (TMB; Kirkegard & Perry, Gaithersburg, MD). Absorbance values at 450 nm were plotted for each dilution; 3 points were chosen from the linear parts of the IgA and IgG standard curves and regression lines were calculated. Two points were then chosen from the linear part of the specimen curves within the range of the regression lines, and IgA and IgG concentrations in the cervical samples were extrapolated.

### Enhanced Chemiluminescence (ECL)-Western Blot

Western blots of polyvinylidine difluoride (Immobilon, Millipore Corp., Bedford, MA) were prepared as described previously[13] using HSV-2 or mocked-infected extracts from conventional cultures of human diploid fibroblasts. The cervical secretions were diluted 1:40 in a 4% solution of goat serum in PBS, then incubated with blots at room temperature overnight on a rocker platform. The blots were washed 3 times with 0.5% Tween-20 in PBS and once with PBS and incubated for 90 min at room temperature with horseradish peroxidase-conjugated secretory antibody: goat anti-human IgA (α chain diluted 1:10,000 in PBS; Pierce) *or* goat antihuman IgG (γ chain diluted 1:10,000 in PBS; Boehringer Mannheim Biochemicals, Indianapolis, IN).

After being thoroughly washed with PBS/Tween, the blots were incubated for 1 min with a commercial Western blotting detection system based on chemiluminescence as directed by the manufacturer (Amersham, Arlington Heights, IL). The blots were then covered with plastic wrap and exposed to Hyperfilm-ECL (Amersham) for a range of times: 10 sec to 3 min. The film was then developed in a Kodak X-Omat processor.

All specimens from each patient were tested against the same lot of ECL-Western blot strips and in the same immunoblot run. For an accurate comparison of antibody profiles across time points, each set of blots for HSV-specific IgA or IgG was developed for the same length of time. The changes in relative levels of IgA or IgG to HSV were scored if at least 3 major bands showed a marked difference in intensity or if the bands were present in 1 specimen and absent in the other specimens being compared. The reactive bands were identified by migration characteristics as described previously.[22,23]

### Statistical Analysis

The median levels of protein and total IgG and IgA from the cervix at each visit were compared using the 2-tailed Wilcoxon's signed-rank test.[24] The changes scored as increased or decreased levels of HSV-specific cervical IgA and IgG by ECL-Western blot between visits were evaluated using a 2-tailed sign test.[24]

### RESULTS

### HSV Serostatus

Of the 37 patients who completed the study, 35 subjects had at least 2 cervical samples taken for local antibody measurements that were Hemoccult negative. Of these, 7 (20%) were HSV seronegative, 14 (40%) had antibodies to HSV-1, 10 (29%) had antibodies to HSV-2, and 4 (11%) had antibodies to both HSV-1 and HSV-2. In the following analyses, the 4 patients with antibodies to both HSV-1 and HSV-2 were grouped with the 10 who had antibodies to HSV-2 only.

### Prenatal Cervical Protein, IgA, and IgG Levels

Thirty-five women had cervical specimens taken during the third trimester for comparison with enrollment samples for total cervical protein concentrations, total IgA, and total IgG. The results according to HSV serostatus are shown in Table 1. No significant differences in median protein, total IgA, or total IgG values were found between the enrollment and third-trimester samples in seronegative women or in HSV-1 or HSV-2 seropositive women compared with HSV seronegative women. Moreover, all values were similar between the 2 time points.

### Postpartum Changes in Total Protein, Total IgA, and IgG

Nineteen of 35 (54%) women had evaluable cervical specimen pairs from third-trimester and postpartum visits (Table 1). Overall, the median values of total protein, IgA, and IgG rose 3-fold to 5-fold in the postpartum samples. This rise was especially apparent in local IgA which rose from a median of 1.32 to 6.3 meg/ml ($P < 0.005$). Of interest, this rise was seen mainly in the HSV seronegative and HSV-1 seropositive subsets. The median IgA values rose approximately 10-fold in seronegative women ($P = 0.08$) and HSV-1 seropositive women ($P = 0.03$) and by 2-fold in HSV-2 seropositive women ($P = 0.35$) (Table 1).

The specimen volume, our measure of adequacy and uniformity of the sampling technique, showed no significant change between the pre- and postpartum samples ($P = 0.50$, $P = 0.51$, $P = 0.35$, and $P = 0.63$ for HSV seronegative, HSV-1 seropositive, HSV-2 seropositive, and all patients, respectively) or between the HSV seronegative and HSV-1 vs. HSV-2 seropositive specimens.

### Enrollment and Third-Trimester Cervical HSV-1 IgA Profiles

Fourteen women who were seropositive for HSV-1 had samples taken both at the enrollment visit and during the third trimester for comparison of HSV cervical antibody profiles. Of these sample pairs, 3 had no detectable IgA to HSV-1 in either sample. Of the 11 with detectable antibodies, 5 had unchanging profiles of HSV-1 cervical IgA and 3 had increases and 3 had decreases in the number and intensity of reactive bands on ECL-Wesetern blot (Table 2). An example of the low prenatal levels of cervical IgA to HSV is shown in Figure 1A (lanes 1 and 2). Note that detectable cervical IgG to HSV was present at all sampling points (Fig. 1B). The serum IgA (Fig. 1C) and IgG antibody

TABLE 1. Cervical protein, IgA, and IgG levels at each visit[a]

| HSV status | No. of patients | Visit | Median protein (range), μg/μl | Median IgA (range), μg/μl | Median IgG (range), μg/μl |
|---|---|---|---|---|---|
| Negative | 7 | Enrollment | 19 (5–53) | 2.23 (0.12–5.54) | 2.78 (0–23.31) |
| | | 3rd trimester | 40 (10–51) | 1.32 (0–4.05) | 2.41 (0–5.76) |
| | | | $P = 0.35$ | $P = 0.18$ | $P = 0.50$ |
| | 5 | 3rd trimester | 24 (10–51) | 1.87 (0–4.05) | 3.41 (0–3.11) |
| | | Postpartum | 250 (30–308) | 12.16 (0.05–45.15) | 14.33 (0.03–21.34) |
| | | | $P = 0.07$ | $P = 0.08$ | $P = 0.08$ |
| HSV-1 only | 14 | Enrollment | 22.5 (4–230) | 1.87 (0.01–19.61) | 3.31 (0.1–36.49) |
| | | 3rd trimester | 29 (10–134) | 1.44 (0.01–7.26) | 3.42 (0.16–18.42) |
| | | | $P = 0.92$ | $P = 0.73$ | $P = 0.93$ |
| | 9 | 3rd trimester | 29 (10–134) | 1.59 (0.32–7.26) | 3.35 (0.83–18.42) |
| | | Postpartum | 62 (13–405) | 10.86 (1.76–24.65) | 10.5 (0.36–16.88) |
| | | | $P = 0.14$ | $P = 0.03$ | $P = 0.17$ |
| HSV-2 | 14 | Enrollment | 25 (0–63) | 1.83 (0.03–12.71) | 4.14 (0.16–41.09) |
| | | 3rd trimester | 22.5 (0–484) | 1.51 (0.03–10.12) | 2.82 (0.252–33.79) |
| | | | $P = 0.68$ | $P = 0.22$ | $P = 0.70$ |
| | 5 | 3rd trimester | 34 (11–114) | 0.97 (0.22–7.56) | 8.47 (1.17–53.79) |
| | | Postpartum | 24 (15–90) | 2.18 (0.27–9.59) | 8.62 (3.65–19.08) |
| | | | $P = 0.69$ | $P = 0.35$ | $P = 0.89$ |
| Total | 35 | Enrollment | 21 (0–230) | 1.93 (0.01–19.61) | 3.49 (0–41.09) |
| | | 3rd trimester | 29 (0–484) | 1.41 (0–10.12) | 3.11 (0–33.79) |
| | | | $P = 0.55$ | $P = 0.11$ | $P = 0.49$ |
| | 19 | 3rd trimester | 29 (10–134) | 1.32 (0–7.56) | 2.72 (0–33.79) |
| | | Postpartum | 91 (13–405) | 6.27 (0.05–45.15) | 10.5 (0.03–31.34) |
| | | | $P = 0.05$ | $P < 0.005$ | $P = 0.08$ |

[a]Wilcoxon's signed-rank test, 2-tailed P value comparing paired visits as indicated.

TABLE 2. Changes in ECL-Western blot cervical antibody profiles against HSV proteins between visits[a]

| HSV serologic status | Cervical IgA | | | | Cervical IgG | | | |
|---|---|---|---|---|---|---|---|---|
| | No change | Increase | Decrease | Total | No change | Increase | Decrease | Total |
| Enrollment and 3rd trimester | | | | | | | | |
| HSV-1 | 5 (45%) | 3 (27%) | 3 (27%) | 11 | 7 (50%) | 3 (21%) | 4 (29%) | 14 |
| HSV-2 | 2 (20%) | 2 (20%) | 6 (60%) | 10 | 4 (29%) | 6 (42%) | 4 (29%) | 14 |
| Total | 7 (33%) | 5 (24%) | 9 (43%) | 21[b] | 11 (39%) | 9 (32%) | 8 (29%) | 28 |
| 3rd trimester and postpartum | | | | | | | | |
| HSV-1 | 0 | 9 (90%) | 1 (10%) | 10 | 1 (10%) | 8 (80%) | 1 (10%) | 10 |
| HSV-2 | 0 | 5 (83%) | 1 (17%) | 6 | 0 | 4 (67%) | 2 (33%) | 6 |
| Total | 0 | 14 (88%) | 2 (12%) | 16* | 1 (6%) | 12 (75%) | 3 (19%) | 16** |

[a]ECL-Western blot profiles of cervical IgA and cervical IgG were compared between enrollment and 3rd-trimester blots and between 3rd-trimester and postpartum blots, as illustrated in Figures 1 and 2. Numbers in parentheses are the percentages of subjects with a given result within the population of pairs scored ("Total"). Increase = at least 3 major bands on ECL-Western blot were markedly increased in intensity or were present in the second but not the first blot of the pair. Decrease = at least 3 major bands on ECL-Western blot were markedly decreased in intensity or were present on the first but not the second blot of the pair. All blots for the same subject were run at the same time under the same conditions for each antibody type (IgA or IgG).

[b]Does not include 3 HSV-1 seropositive and 4 HSV-2 seropositive women who either had no detectable IgA antibody to HSV in their samples or <10 ng of protein in one or both samples.

*$P = 0.004$ by 2-tailed sign test.

**$P = 0.08$ by 2-tailed sign test.

profiles to HSV-1 (Fig. 1D) did not change over time. No cervical IgA or IgG antibodies against HSV-1 or HSV-2 proteins were detected at any of the 3 visits in seronegative patients.

Five of the 6 women who had apparent changes in cervical IgA profiles had concordant changes (either increases or decreases) in their total protein levels as well. The volumes of the paired specimens in 5 of

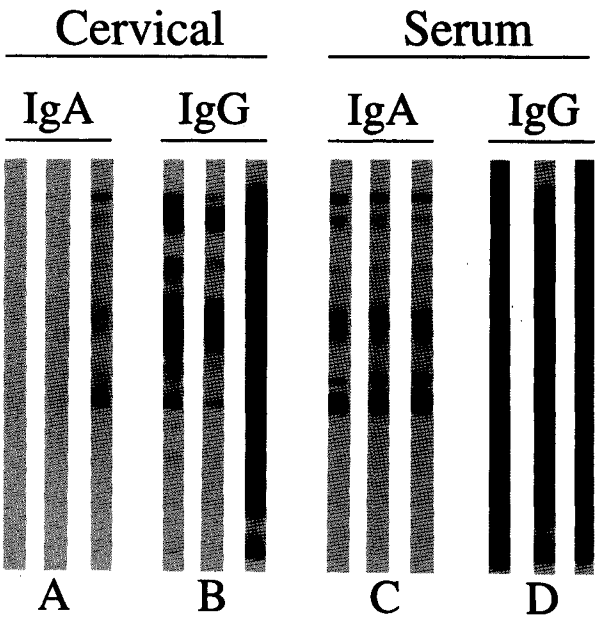

Fig. 1. Cervical and serum antibody profiles from samples obtained over time from an HSV-1 seropositive patient. ECL-Western blots were performed to detect cervical antibodies to HSV-1 **(A,B)** and serum antibodies to HSV-1 **(C,D)**. The left blot in each panel is from specimens taken at enrollment, the middle blot from specimens taken during the third trimester, and the right blot from specimens taken 6 weeks postpartum. Note the dramatic increase in band number and intensity in cervical IgA and IgG to HSV-1 between the third-trimester and postpartum samples. In contrast, the serum profiles show no change in the IgA profile to HSV-1 over the 3 time points. The serum IgG profiles, while difficult to read at this exposure because of the high IgG, show evidence of a decrease in intensity in the third-trimester serum and recovery of intensity in the postpartum serum.

the 6 pairs with changes were either similar between the pairs or showed discordant change, suggesting that sampling differences alone were not sufficient to explain the changes. The sixth pair had a lower volume in the third-trimester sample along with decreasing apparent levels of IgA to HSV-1; however, her IgG levels increased between the 2 specimens. Of note was that 1 subject had labial blisters that were culture positive for HSV-1 at the time of her third-trimester sampling; her HSV-1-specific IgA in genital secretions decreased to undetectable levels at this sampling. None of the other genital cultures obtained were positive for HSV during the study.

### Postpartum Cervical HSV-1 IgA

Fourteen women with HSV-1 antibody had specimens taken in the third trimester and at 6 weeks postpartum. The postpartum samples from 4 women contained blood and could not be evaluated for cervical antibody. Of the 10 available pairs, all had demonstrable HSV-IgA in the postpartum cervical secretions and 9 (90%) had clear increases in cervical HSV-IgA profile complexity and intensity, while 1 had a decrease (Table 2). Figure 1A (lane 3) gives an example of the increased intensity and complexity of the IgA profile postpartum.

### Enrollment and Third-Trimester Cervical HSV-2 IgA Profiles

IgA to HSV-2 was compared in enrollment and third-trimester samples of 14 women who were seropositive for HSV-2. Of the 14 pairs, 4 lacked detectable IgA. Of the 10 with detectable HSV-2 antibody, 2 had no change, 2 had increasing complexity and intensity of profiles, and 6 had decreasing amounts of antibodies in their cervical secretions (Table 2). An example of a decrease in cervical IgA band number and intensity is shown in Figure 2A (lanes 1 and 2). This patient also had a decrease in cervical IgG in the third-trimester sample (Fig. 2B, lanes 1 and 2).

### Postpartum Cervical HSV-2 IgA

Of the 14 HSV-2 seropositive women, 9 had specimens taken at 6 weeks postpartum. Three specimens were not used for comparison of antibody profiles because they contained blood. Five of the 6 (83%) available pairs showed clear increases in IgA complexity and intensity in the postpartum samples and the sixth showed a decrease (Table 2). The patient in Figure 2A (lane 3) exemplifies the dramatic changes in cervical IgA profiles postpartum.

Overall, in 21 evaluable subjects with detectable cervical IgA to HSV-1 or HSV-2, 43% had decreases in apparent antibody levels between enrollment and the third trimester; 24% had increases; and 33% had no apparent change. Overall, 14 of 16 (88%) HSV seropositive women had postpartum increases in their cervical IgA to HSV ($P = 0.004$ by 2-tailed sign test).

### Enrollment and Third-Trimester Cervical HSV IgG

All 28 patients with paired specimens from enrollment and third-trimester visits had detectable cervical anti-HSV IgG. Among the 14 with HSV-1 anti-

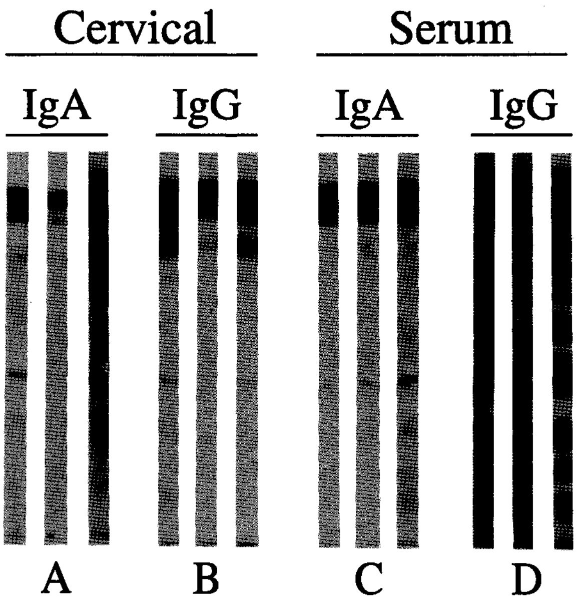

Fig. 2. Cervical and serum antibody profiles in samples obtained over time from an HSV-2 seropositive patient. ECL-Western blots were performed to detect cervical antibodies to HSV-2 **(A,B)** and serum antibodies to HSV-2 **(C,D).** The left blot in each panel is from specimens taken at enrollment, the middle blot from specimens taken during the third trimester, and the right blot from specimens taken 6 weeks postpartum. Note the dramatic change in intensity and number of bands in the cervical IgA profile of the postpartum specimen compared with the cervical IgA profiles from specimens taken during pregnancy. In contrast, the cervical IgG and serum IgA and IgG profiles changed little over time.

body, 7 had no change in IgG profiles, 3 had increased intensity of their profiles, and 4 had decreased intensity of their profiles at the third trimester (Table 2). Of the 14 HSV-2 seropositive patients, 4 had no change in cervical IgG profiles, 6 had increased intensity of reactive bands, and 4 had decreased intensity of their profiles (Table 2). Overall, of the 28 patients, 39% had no change in cervical IgG, 32% had increased apparent levels of cervical IgG, and 29% had decreased intensity of bands at the third trimester.

### Postpartum Cervical HSV IgG

Eight of 10 (80%) pairs of specimens from HSV-1 seropositive women showed pronounced increases in cervical IgG to HSV-1 in the postpartum samples (Table 2). Figure 1B (lane 3) shows a modest increase in cervical IgG postpartum. In 8 patients, the IgG changes were concordant with IgA changes. The IgA increased while the IgG showed no change postpartum in 1 patient and the IgA increased while the IgG decreased in 1 patient. Four of 6 (67%) available pairs had pronounced increases in cervical IgG to HSV-2, while 2 (33%) had decreases. The changes in cervical HSV-2 antibodies were concordant between IgA and IgG in 5 of the 6 pairs; the sixth had an increase in IgA and a decrease in IgG postpartum. Overall, 12 patients (75%) had increased cervical IgG to HSV postpartum, while 3 (19%) had decreases and 1 (6%) had no change ($P = 0.08$ for proportion with increased postpartum IgG by 2-tailed sign test).

### Serum Antibody Profiles Pre- and Postpartum

Sera were drawn at all 3 visits from 6 HSV-1 and 6 HSV-2 seropositive women. Serum IgA and IgG profiles were examined between enrollment and the third-trimester and postpartum visits. Among the 6 women with HSV-1 antibodies, 3 had no detectable changes, 2 had IgA (but not IgG) profiles that decreased in complexity in the third trimester and recovered to enrollment levels postpartum, and a third had IgG (but not IgA) levels that dropped at the third trimester but recovered postpartum. In this latter patient, the cervical IgG changes roughly paralleled those of serum IgG (Fig. 1B vs. Fig. 1D), while the cervical IgA changed markedly without a concomitant change in serum IgA profiles (Fig. 1A vs. Fig. 1C). Of the 6 who were HSV-2 seropositive, 3 had no detectable changes, 1 had a rise in serum IgA (but not IgG) at the third trimester, 1 had a decrease in both serum IgA and IgG at the third trimester (changes which were reflected in similar changes in cervical antibody), and 1 had a possible rise in serum IgA in the postpartum sample (Fig. 2C). Overall, of the 12 women with sequential serum samples, the changes in apparent levels of serum HSV-specific IgA and IgG over time were either not detected or were minor compared with the profile changes seen over time with cervical HSV-specific IgA and IgG.

### DISCUSSION

This pilot study indicates that there are fluctuations in HSV-specific antibodies in cervical secretions that are not seen in serum. Although serum antibody profiles changed little between pregnancy and the postpartum visits, the total protein, total IgA, and HSV-specific antibody levels in the cervix increased postpartum. The increasing intensity and complexity of cervical HSV antibody profiles, both IgA and IgG, at the postpartum visit are striking. It is tempting to assume that these relatively low levels of cervical antibodies late in pregnancy reflect broader immune suppression, which may account for the previously reported increased rate of positive genital herpes cultures during the third trimester.[10] However, a study of more women earlier in pregnancy and for a longer period postpartum is necessary to evaluate whether the increasing levels seen postpartum are the normal levels or whether they are transient evaluations. In previous studies of nonpregnant women with either first-episode or recurrent genital HSV infections, the cultures were not positive when the cervical IgA antibody level was ≥1:2.[17] In addition, the mean duration of genital shedding of HSV with both first-episode and recurrent outbreaks was 3 days shorter among women who developed secretory IgA compared with those without IgA.[17] These and more recent studies[19] are suggestive that IgA may play a role in limiting the duration of HSV shedding. However, since only 1 patient had a positive culture at any visit in our study, only concurrent, more frequent sampling for both virus and antibody in the cervix could address this question.

The lack of change in serum IgG and IgA antibody levels over the course of pregnancy and the postpartum visit is consistent with previous studies of antibody levels in pregnant women.[20] The serum antibody patterns do not account for the observed changes in cervical antibody levels. Although no changes were seen in the cervical levels of total protein, IgG, or IgA between the enrollment and third-trimester sampling points, the enrollment visit occurred anytime before 20 weeks gestation. The changes related to pregnancy may occur early in pregnancy, but our sampling protocol did not include first-trimester visits in all cases. One important finding of our study was the clear increase in total cervical IgA levels between the third-trimester and postpartum sampling. However, the total cervical IgG levels did not change significantly from the third-trimester to the postpartum sampling.

It is not clear why the increase in cervical total IgA and HSV-specific IgA was only 2-fold in HSV-2 positive patients compared with 6-fold in HSV-1 only or HSV antibody negative women. It did not appear to be related to the sampling technique. The volume as measured in each sample was similar at all 3 time points and in all 3 serologic groups, but only a small number of women were studied. Differences in HSV reactivation in the genital tract may be a factor influencing this observation. Our test method could not detect antibodies that were complexed with antigen. Any apparent changes in HSV-2 cervical antibodies may be affected by viral shedding. More detailed study of changes in cervical antibodies during and after pregnancy among women with genital HSV-1 infections would help to elucidate whether the specific type of HSV infecting the genital area has an effect on local antibody levels during and after pregnancy. Such studies will require more patients, more frequent sampling, and more quantitative testing methods.

The differences in local antibodies seen during pregnancy compared with postpartum values may be related to the hormone changes of pregnancy. Of interest, the serum antibody levels in pregnant women do not show significant changes during pregnancy compared with postpartum. In a study of secretory component production by uterine tissues in ovariectomized rats, progesterone was found to cause a marked decrease in the production of secretory component.[25] This effect, which was dose dependent, occurred whether or not the animals were pretreated with estradiol. The high levels of progesterone present during pregnancy may inhibit the production of secretory component which would then limit the local IgA antibody levels. These factors could be further evaluated by more frequent sampling of cervical IgA levels throughout pregnancy and pairing them with progesterone levels since the concentration of progesterone increases markedly over the course of pregnancy. If progesterone specifically affects secretory component and not overall immunoglobulin production, it would account for differences in local antibodies without concomitant changes in serum antibody levels.

For the evaluation of the changes in local immunity suggested by this pilot study, a more comprehensive and detailed study is required. Comparing a control group of nonpregnant women sampled at similar intervals with a group of women before, during, and after pregnancy would allow an evaluation of the changes over time not related specifically to pregnancy. More frequent sampling for both local antibody levels and HSV reactivation detected by culture and polymerase chain reaction should be included to assess the relationship between local immunity and HSV shedding. In addition, a measurement of antibody levels to an antigen not present in the genital tract such as tetanus would allow a delineation of the changes in levels related to antigenic stimulation compared with nonspecific immune-system activation or changes related to pregnancy. The development of more precise methods for collection and quantitation of HSV-specific antibody levels in cervical secretions rather than just a comparison of changes in intensity and complexity of Western blot patterns is necessary to evaluate potential pregnancy-related changes in local antibody. Furthermore, since Western blots measure antibody levels to denatured proteins rather than the intact virion, a correlation of antibody levels with neutralizing activity, viral shedding, and cellular immune responses in the genital tract is necessary to evaluate the clinical significance of changes in ECL-Western blot antibody responses over time.

The methods used in our study could be adapted for a study of the mucosal immune responses to other antigens as well. An evaluation of genital immunity, including antibody response, will be crucial in evaluating the response to human immunodeficiency virus, especially to vaccines designed to protect against human immunodeficiency virus infection.

### ACKNOWLEDGMENTS

This work was supported by NIH grants AI 30731-01A1 and NIH PO1 AI29363 from the National Institute of Allergy and Infectious Diseases.

### REFERENCES

1. Koutsky LA, Stevens CE, Holmes KK, et al.: Underdiagnosis of genital herpes by current clinical and viral-isolation procedures. N Engl J Med 326:1533–1539, 1992.
2. Frenkel LM, Garratty EM, Shen JP, et al.: Clinical reactivation of herpes simplex virus type 2 infection in seropositive pregnant women with no history of genital herpes. Ann Intern Med 119:414, 1993.
3. Bryson Y, Dillon M, Bernstein DI, et al.: Risk of acquisition of genital herpes simplex virus type 2 in sex partners of persons with genital herpes: A prospective couple study. J Infect Dis 167:942–946, 1993.
4. Kulhanjian JA, Soroush V, Au DS, et al.: Identification of women at unsuspected risk of primary infection with herpes simplex virus type 2 during pregnancy. N Engl J Med 326:916–920, 1992.
5. Brown ZA, Benedetti J, Ashley R: Neonatal herpes simplex virus infection in relation to asymptomatic maternal infection at the time of labor. N Engl J Med 324:1247–1252, 1991.
6. Ashley RL, Dalessio J, Burchett S: Herpes simplex virus-2 (HSV-2) type-specific antibody correlates of protection in infants exposed to HSV-2 at birth. J Clin Invest 90:511–514, 1992.
7. Prober CG, Sullender WM, Yasukawa LL, et al.: Low risk of herpes simplex infections in neonates exposed to the virus at the time of vaginal delivery to mothers with recurrent genital herpes simplex virus infections. N Engl J Med 316:240–244, 1987.
8. Whitley RJ: Herpes simplex virus infections. In Remington J, Klein J (eds): Infectious Diseases of the Fetus and Newborn. Philadelphia: W.B. Saunders, pp 282–305, 1990.
9. Sullender WM, Yasukawa LL, Schwartz M, et al.: Type-specific antibodies to herpes simplex virus type 2 (HSV-2) glycoprotein G in pregnant women, infants exposed to maternal HSV-2 infection at delivery and infants with neonatal herpes. J Infect Dis 157:164–171, 1988.
10. Brown ZA, Vontver LA, Benedetti J: Genital herpes in pregnancy: Risk factors associated with recurrences and asymptomatic viral shedding. Am J Obstet Gynecol 153:24–30, 1985.
11. Baker DA, Thomas J, Epstein J, Possilico D, Stone ML: The effect of prostaglandins on the multiplication and cell-to-cell spread of herpes simplex virus type 2 in vitro. Am J Obstet Gynecol 144:346–349, 1982.
12. Weinberg ED: Pregnancy-associated depression of cell-mediated immunity. Rev Infect Dis 6:814, 1984.
13. Coughlan BM, Skinner GRB: Antibody activity to type 1 and type 2 herpes simplex virus in human cervical mucus. Br J Obstet Gynaecol 84:622–629, 1977.
14. Gonroos M, Honkonen E, Terho P, Punnonen R: Cervical and serum IgA and serum IgG antibodies to *Chlamydia trachomatis* and herpes simplex virus in threatened abortion: A prospective study. Br J Obstet Gynaecol 90:167–170, 1983.
15. Murphy JF, Murphy DF, Barker S, Mylotte ML, Coughlan BM, Skinner GRB: Neutralizing antibody against type 1 and type 2 herpes simplex virus in cervical mucus of women with cervical intraepithelial neoplasia. Med Microbiol Immunol 174:73–80, 1985.
16. Persson E, Eneroth P, Jeansson S: Secretory IgA against herpes simplex virus in cervical secretions. Genitourin Med 64:373–377, 1988.
17. Merriman H, Woods S, Winter C, Fahnlander A, Corey L: Secretory IgA antibody in cervicovaginal secretions from women with genital infection due to herpes simplex virus. J Infect Dis 149:505–510, 1984.
18. Ashley RL, Corey L, Dalessio J: Protein-specific cervical antibody responses to primary genital herpes simplex virus type 2 infections. J Infect Dis 170:20–26, 1994.
19. Ashley R, Wald A, Corey L: Cervical antibodies in patients with oral herpes simplex virus type 1 (HSV-1) infection. Local anamnestic response after genital HSV-2 infection. J Virol 68:5284–5286, 1994.

20. Ostenson M, Lundgren R, Husby G, Rikorg OP: Studies on humoral immunity in pregnancy: Immunoglobulins, alloantibodies, and autoantibodies in healthy pregnant women and in pregnant women with rheumatoid disease. J Clin Lab Immunol 11:143–147, 1983.
21. Ashley RL, Corey L: Effect of acyclovir treatment of primary genital herpes on the antibody response to herpes simplex virus. J Clin Invest 73:681–688, 1984.
22. Dalessio J, Ashley RL: Highly sensitive enhanced chemiluminescence immunodetection method for herpes simplex virus type 2 Western immunoblot. J Clin Microbiol 30:1005–1007, 1992.
23. Ashley RL, Mack K, Critchlow C, Shurtleff M, Corey L: Different effect of systemic acyclovir treatment of genital HSV-2 infections on antibody responses to individual HSV-2 proteins. J Med Virol 24:309–319, 1988.
24. Snedecor GW, Cochran WG; Statistical Methods. 7th ed. Ames: Iowa State University Press, pp 135–148, 1980.
25. Stern JE, Wira CR: Progesterone regulation of secretory component (SC): Uterine SC response in organ cultures following in vivo hormone treatment. J Steroid Biochem 30:233–237, 1988.
